# Phenotypes and *PRRT2* mutations in Chinese families with benign familial infantile epilepsy and infantile convulsions with paroxysmal choreoathetosis

**DOI:** 10.1186/1471-2377-13-209

**Published:** 2013-12-26

**Authors:** Xiaoling Yang, Yuehua Zhang, Xiaojing Xu, Shuang Wang, Zhixian Yang, Ye Wu, Xiaoyan Liu, Xiru Wu

**Affiliations:** 1 Department of Pediatrics, Peking University First Hospital, No. 1 of Xian Men Street, , Beijing, Xicheng District 100034, China

**Keywords:** Benign familial infantile epilepsy, Infantile convulsions with paroxysmal choreoathetosis, Phenotype, *PRRT2*, Mutation

## Abstract

**Background:**

Mutations in the *PRRT2* gene have been identified as the major cause of benign familial infantile epilepsy (BFIE), paroxysmal kinesigenic dyskinesia (PKD) and infantile convulsions with paroxysmal choreoathetosis/dyskinesias (ICCA). Here, we analyzed the phenotypes and *PRRT2* mutations in Chinese families with BFIE and ICCA.

**Methods:**

Clinical data were collected from 22 families with BFIE and eight families with ICCA. *PRRT2* mutations were screened using PCR and direct sequencing.

**Results:**

Ninety-five family members were clinically affected in the 22 BFIE families. During follow-up, two probands had one seizure induced by diarrhea at the age of two years. Thirty-one family members were affected in the eight ICCA families, including 11 individuals with benign infantile epilepsy, nine with PKD, and 11 with benign infantile epilepsy followed by PKD. Two individuals in one ICCA family had PKD or ICCA co-existing with migraine. One affected member in another ICCA family had experienced a fever-induced seizure at 7 years old. *PRRT2* mutations were detected in 13 of the 22 BFIE families. The mutation c.649_650insC (p.R217PfsX8) was found in nine families. The mutations c.649delC (p.R217EfsX12) and c.904_905insG (p.D302GfsX39) were identified in three families and one family, respectively. *PRRT2* mutations were identified in all eight ICCA families, including c.649_650insC (p.R217PfsX8), c.649delC (p.R217EfsX12), c.514_517delTCTG (p.S172RfsX3) and c.1023A > T (X341C). c.1023A > T is a novel mutation predicted to elongate the C-terminus of the protein by 28 residues.

**Conclusions:**

Our data demonstrated that *PRRT2* is the major causative gene of BFIE and ICCA in Chinese families. Site c.649 is a mutation hotspot: c.649_650insC is the most common mutation, and c.649delC is the second most common mutation in Chinese families with BFIE and ICCA. As far as we know, c.1023A > T is the first reported mutation in exon 4 of *PRRT2*. c.649delC was previously reported in PKD, ICCA and hemiplegic migraine families, but we further detected it in BFIE-only families. c.904_905insG was reported in an ICCA family, but we identified it in a BFIE family. c.514_517delTCTG was previously reported in a PKD family, but we identified it in an ICCA family. Migraine and febrile seizures plus could co-exist in ICCA families.

## Background

Benign familial infantile epilepsy (BFIE), formerly called benign familial infantile seizures (OMIM 605751), is a benign familial focal epilepsy syndrome [1,2]. It is characterized by afebrile seizures with onset between 3 and 12 months of age. Seizures are partial, with or without secondary generalization, often occur in clusters and usually remit before 2 years of age. During childhood or adolescence, family members in BFIE may develop paroxysmal kinesigenic choreoathetosis or dyskinesias (PKC/D). The term “infantile convulsions with paroxysmal choreoathetosis” (ICCA, OMIM 602066) has been used to describe the phenotype of infantile convulsions and paroxysmal dyskinesias (choreoathetosis or dystonia) co-occurring in the same patient or family [3]. Paroxysmal kinesigenic dyskinesia (PKD, OMIM128200) was first described by Demirkiran, who suggested using the generic term “dyskinesia”, rather than dystonia, chorea, or choreoathetosis [4]. *PRRT2* gene, encoding proline-rich transmembrane protein 2, was recently identified as a major causative gene for BFIE, PKD and ICCA [5-8]. The aim of this study was to analyze the clinical features and *PRRT2* mutations in Chinese families with BFIE and ICCA.

## Methods

### Patients

This study was approved by the Ethics Committee of Peking University First Hospital. Written informed consent for publication of their clinical details was obtained from the patients or their parents in case of minors. We recruited 22 BFIE families and eight ICCA families with autosomal dominant inheritance at Peking University First Hospital from September 2006 to July 2013. Clinical information was collected from the probands and their family members.

The diagnostic criteria for BFIE were as follows [9,10]: (1) seizure onset between 3 and 12 months; (2) seizures in clusters; (3) focal seizures with or without secondary generalization, usually manifesting motor arrest, deviation of the head and eyes to one side, generalized hypertonia, cyanosis, and limb jerks; (4) the interictal electroencephalography (EEG) is normal while the ictal EEG shows abnormalities that may originate from various cerebral lobes; (5) normal brain imaging; (6) normal psychomotor development before, during and after the onset of seizures; (7) family history of seizures (similar age at onset); (8) good response to treatment and seizures remitted often before the age of 2 years. A diagnosis of PKD was determined according to the criteria proposed by Bruno as follows [11]: (1) age at onset between1 and 20 years; (2) identified kinesigenic trigger for the attacks; (3) short duration of attacks (<1 minute); (4) no loss of consciousness or pain during attacks; (5) control of attacks with phenytoin or carbamazepine; (6) exclusion of other organic diseases and normal neurologic examination. The diagnosis of ICCA was considered according to Szepetowski if the two clinical manifestations of BFIE and PKD were present in the same patient or different family members [3].

### Genetic analysis

Blood samples were obtained from the probands and their family members where possible. Genomic DNA was extracted from peripheral blood by standard protocols. Mutation screening of *PRRT2* was performed using PCR and direct sequencing. Primers for *PRRT2* (NM_145239.2) were designed using Primer Premier 5.0 software. The three coding exons (containing the coding sequence of the three isoforms of the protein), 5′ untranslated region (containing exon 1), and their flanking introns of the *PRRT2* gene were sequenced. Primer sequences and annealing temperatures for PCR are available upon request. Mutations found in a proband were examined for co-segregation in other family members. The *PRRT2* mutations found in the patients were also screened in 100 Chinese unrelated healthy controls. Impact of an amino acid substitution of missense mutation was predicted by PolyPhen-2 (http://genetics.bwh.harvard.edu/pph2/). Patients were followed up at a pediatric neurology clinic at our hospital or by telephone.

## Results

### Clinical findings

#### ***BFIE families***

**Figure 1:**
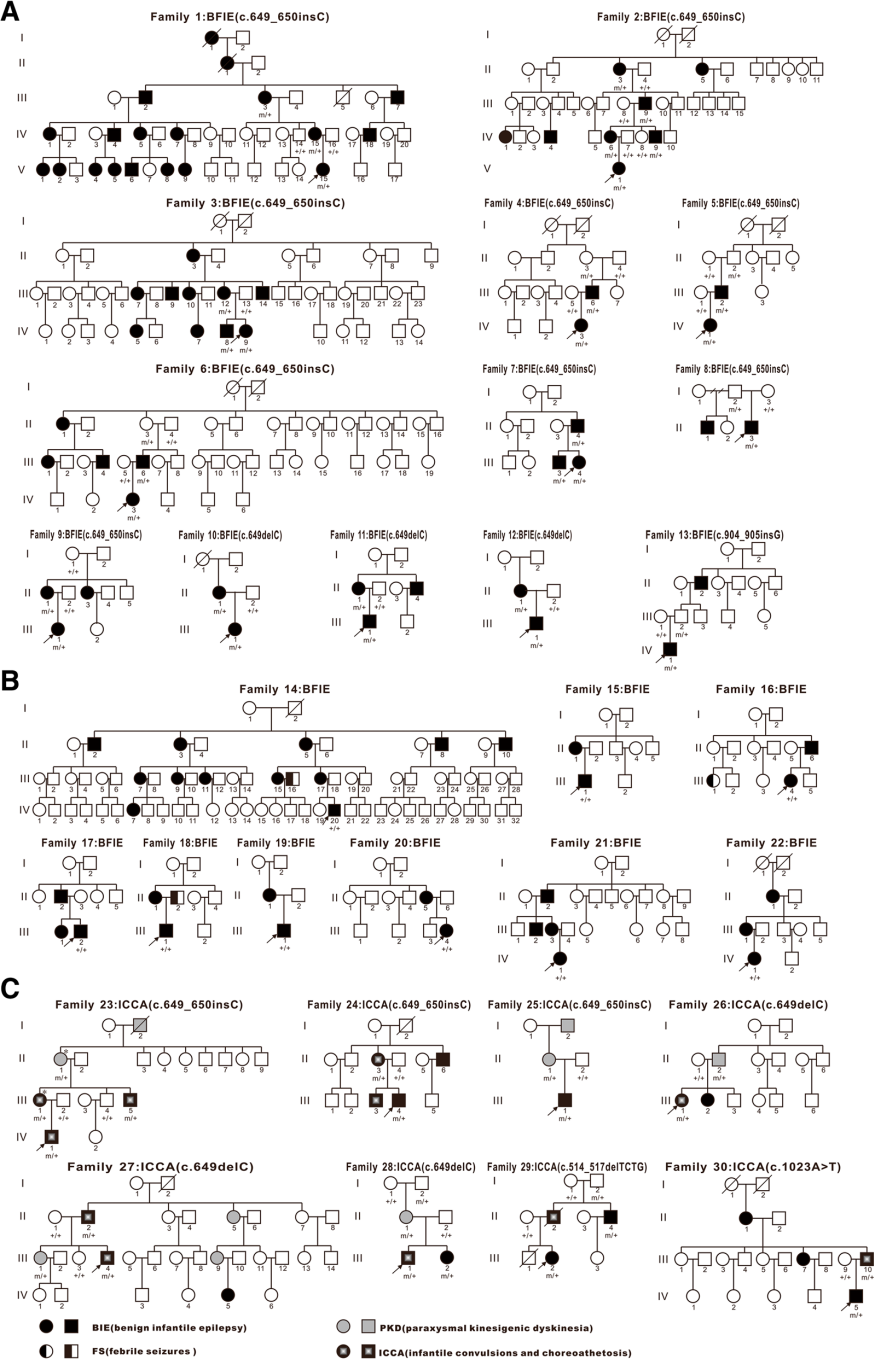
**Pedigrees of the 22 BFIE families and 8 ICCA families.** The arrow indicates the proband. An individual with a heterozygous *PRRT2* mutation is indicated by m/+, and an individual without a *PRRT2* mutation is indicated by +/+. An individual with an asterisk is a patient with migraine. **(A)** Pedigrees of families 1-13: thirteen BFIE families with a *PRRT2* mutation, **(B)** Pedigrees of families 14-22: nine BFIE families without a *PRRT2* mutation, **(C)** Pedigrees of families 23-30: eight ICCA families with a *PRRT2* mutation.

Among the 22 families with BFIE, 95 family members were affected. Families with at least two members affected by benign infantile epilepsy (BIE) were included and the largest one had 19 affected members over five generations (Family 1). Family pedigrees of BFIE families with a *PRRT2* mutation are shown in Figure 1A and pedigrees of BFIE families without a *PRRT2* mutation are shown in Figure 1B. Among the 22 probands, the age of seizure onset was 3–11 months (median: 4.5 months). Seizures remitted either spontaneously or after treatment with antiepileptic drugs. However, two probands of unrelated families had one afebrile seizure induced by diarrhea at the age of 2 years. The age at last follow-up of these two probands was 2 years and 9 months (Family 6: IV-3) and 3 years and 2 months (Family 11: III-1), respectively. In BFIE Family 6, the proband (IV-3) had seizure onset at 4.5 months of age and spontaneous remission at the age of 6 months without antiepileptic treatment, but she had one afebrile seizure at 31 months of age induced by diarrhea, manifesting as eye deviation to the left side without loss of consciousness, which lasted about one minute. In BFIE Family 11, the proband (III-1) had seizure onset at 3 months of age and remission after treatment with levetiracetam. He had one afebrile generalized tonic-clonic seizure at 25 months of age in conjunction with diarrhea. The interictal EEG of these two probands was normal.

The clinical features of all the BFIE probands and the detected *PRRT2* mutations are summarized in Table 1. The clinical data of other affected members in BFIE families were also collected but not included in the Table 1, because for some, a detailed clinical history of infantile seizures could not be obtained. The clinical history of the other 73 affected relatives were shown in Additional file 1: Table S1. Families 1–13 were *PRRT2* mutation-positive and Families 14–22 were *PRRT2* mutation-negative.

**Table 1:** **Clinical features and *PRRT2 *** **mutations in the probands of 22 BFIE families**

**Family No.**	**Proband**	**Sex**	**Age of seizure onset**	**Age at last follow-up**	**Nucleotide change**	**Amino acid change**
1	V-15	F	4.5 m	4y5 m	c.649_650insC	p.R217PfsX8
2	V-1	F	5 m	10 m	c.649_650insC	p.R217PfsX8
3	IV-9	F	4 m	1y	c.649_650insC	p.R217PfsX8
4	IV-3	F	4.5 m	2y5m	c.649_650insC	p.R217PfsX8
5	IV-1	F	7 m	1y2m	c.649_650insC	p.R217PfsX8
6	IV-3	F	4.5 m	2y9m	c.649_650insC	p.R217PfsX8
7	III-4	F	3.5 m	4y	c.649_650insC	p.R217PfsX8
8	II-3	M	3.5 m	8 m	c.649_650insC	p.R217PfsX8
9	III-1	F	5 m	4y5m	c.649_650insC	p.R217PfsX8
10	III-1	F	3.5 m	5y8m	c.649delC	p.R217EfsX12
11	III-1	M	3 m	3y2m	c.649delC	p.R217EfsX12
12	III-1	M	4 m	8 m	c.649delC	p.R217EfsX12
13	IV-1	M	6 m	3y	c.904_905insG	p.D302GfsX39
14	IV-20	M	3 m	6y10m	none	none
15	III-1	M	3.5 m	5y11m	none	none
16	III-4	F	6 m	2y11m	none	none
17	III-2	M	4 m	5y7m	none	none
18	III-1	M	4.5 m	3y	none	none
19	III-1	M	3 m	1y	none	none
20	III-4	F	10 m	1y10m	none	none
21	IV-1	F	11 m	2y1m	none	none
22	IV-1	F	8 m	3y7m	none	none

M: male, F: female, y: years, m: months.

#### ***ICCA families***

The clinical features of the affected members in the ICCA families and the detected *PRRT2* mutations are summarized in Table 2. In the eight ICCA families, 31 family members were affected, of which 11 individuals had BIE only, 9 individuals had PKD only, and 11 individuals had BIE followed by PKD. Pedigrees of ICCA families are shown in Figure 1C. In Family 23, the phenotypes of PKD or ICCA and migraine with aura co-existed (II-1 and III-1). In Family 27, the proband had a seizure during fever at 7 years old.

**Table 2:** **Clinical features and *PRRT2 *** **mutations in 31 affected members from 8 ICCA families**

				**Infantile convulsions**		**Paroxysmal dyskinesia**					
**Family No**	**Individual**	**Phenotype**	**Sex**	**Age of seizure onset**	**Age of seizure remission**	**Age at onset**	**Trigger**	**Involuntary movements**	**Age at last follow-up**	**Nucleotide change**	**Amino acid change**
23	I-2	PKD	M	-	-	Adolescence	SM	D	50y*	na	na
	II-1	PKD	F	-	-	10y	SM	D	65y	c.649_650insC	p.R217PfsX8
	III-1	ICCA	F	<12 m	24 m	9y	SM	D	42y	c.649_650insC	p.R217PfsX8
	III-5	ICCA	M	<12 m	24 m	8y	SM	D	38y	c.649_650insC	p.R217PfsX8
	IV-1	ICCA	M	7 m	12 m	10y	SM	D/C	11y	c.649_650insC	p.R217PfsX8
24	II-3	ICCA	F	<12 m	<24 m	7y	SM	D	34y	c.649_650insC	p.R217PfsX8
	II-6	BIE	M	<12 m	<24 m	-	-	-	33y	na	na
	III-3	ICCA	M	6 m	8 m	7y	SM	C	9y	na	na
	III-4	BIE	M	4.5 m	5 m	-	-	-	6 m	c.649_650insC	p.R217PfsX8
25	I-2	PKD	M	-	-	14y	SM	C	51y	na	na
	II-1	PKD	F	-	-	12y	SM	C	29y	c.649_650insC	p.R217PfsX8
	III-1	BIE	M	3.5 m	5 m	-	-	-	5.5 m	c.649_650insC	p.R217PfsX8
26	II-2	PKD	M	-	-	11y	SM	D	42y	c.649delC	p.R217EfsX12
	III-1	ICCA	F	8 m	10 m	8y	SM/S	D	16y	c.649delC	p.R217EfsX12
	III-2	BIE	F	5 m	9 m	-	-	-	14y	na	na
27	II-2	ICCA	M	<12 m	24 m	15y	SM/S	D	50y	c.649delC	p.R217EfsX12
	II-5	PKD	F	-	-	10y	SM	D	42y	na	na
	III-1	PKD	F	-	-	10y	SM	D/C	26y	c.649delC	p.R217EfsX12
	III-4	ICCA	M	5.5 m	12 m	8y	SM/S	D/C	13y	c.649delC	p.R217EfsX12
	III-9	PKD	F	-	-	10y	SM	D	22y	na	na
	IV-5	BIE	F	4.5 m	24 m	-	-	-	3y	na	na
28	II-1	PKD	F	-	-	5y	SM	C	35y	c.649delC	p.R217EfsX12
	III-1	ICCA	M	4 m	4.5 m	5.5y	SM, Ex	C	12y	c.649delC	p.R217EfsX12
	III-2	BIE	F	4 m	5 m	-	-	-	5y	c.649delC	p.R217EfsX12
29	II-2	ICCA	M	4.5 m	12 m	16y	SM	D	40y*	na	na
	II-4	BIE	M	<12 m	<24 m	-	-	-	36y	c.514_517delTCTG	p.S172RfsX3
	III-2	BIE	F	3 m	5 m	-	-	-	5y	c.514_517delTCTG	p.S172RfsX3
30	II-1	BIE	F	<12 m	<24 m	-	-	-	83y	na	na
	III-7	BIE	F	3 m	24 m	-	-	-	50y	na	na
	III-10	ICCA	M	3 m	9 m	12y	SM	D	41y	c.1023A > T	X341C
	IV-5	BIE	M	4 m	8 m	-	-	-	3y9m	c.1023A > T	X341C

PKD: paroxysmal kinesigenic dyskinesias, BIE: benign infantile epilepsy, ICCA: infantile convulsions with paroxysmal choreoathetosis, M: male, F: female, y: years, m: months, -: negative, SM: sudden movement, S: startle, Ex: exercise, D: dystonia, C: choreoathetosis, na: not available, *deceased.

### Genetic analysis

#### ***BFIE families***

**Figure 2:**
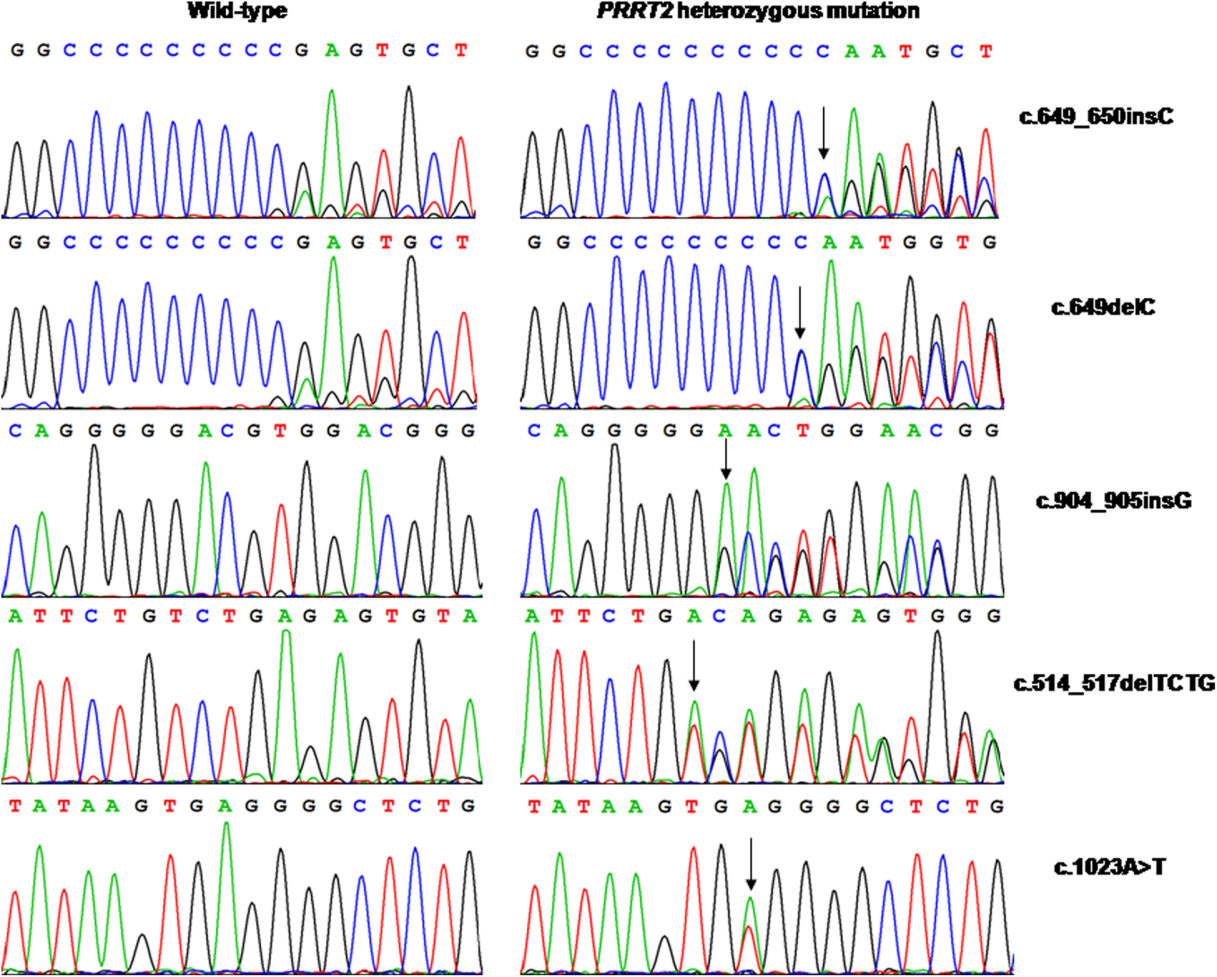
**Sequencing chromatograms showing the five *PRRT2 *mutations detected in BFIE or ICCA families, compared with wild-type traces.** The arrow shows the position of the mutation.

In our 22 BFIE families, three truncating mutations in *PRRT2* were identified in 13 of 22 families (Figure 2), leading to a mutation rate of 59.1%. The mutation c.649_650insC (p.R217PfsX8) was found in nine BFIE families (Families 1–9), accounting for 69.2% (9/13) of BFIE families with a *PRRT2* mutation. In four families, incomplete penetrance was observed (Family 4: II-3, Family 5: II-2, Family 6: II-3, and Family 8: I-2) (Figure 1A). The mutation c.649delC was identified in three BFIE families (Families 10–12). The mutation c.904_905insG (p.D302GfsX39) was detected in one BFIE family (Family 13). In this family, incomplete penetrance was also observed, the proband’s father (III-2) carried the mutation without seizures.

#### ***ICCA families***

In the eight ICCA families, four different *PRRT2* mutations were identified (Figure 2). The mutation c.649_650insC (p.R217PfsX8) was identified in three families, one of whom had ICCA and co-existing migraine (Family 23). The mutation c.649delC (p.R217EfsX12) was found in three families (Families 26–28) and the mutation c.514_517delTCTG (p.S172RfsX3) was present in one family (Family 29). A novel mutation c.1023A > T (X341C) in exon 4 was found in one ICCA family (Family 30). This mutation would result in abrogation of the stop codon and elongation of the peptide by 28 amino acids at the C-terminal. This mutation was not found in public single nucleotide polymorphism databases.

## Discussion

*PRRT2* is an important gene recently identified in neurological paroxysmal disorders. *PRRT2* is the major causative gene of PKD, BFIE, and ICCA [5-8,12-19], and is also responsible for several familial or sporadic cases with paroxysmal non-kinesigenic dyskinesia (PNKD), paroxysmal exercised-induced dyskinesia (PED), sporadic BIE, and hemiplegic migraine (HM) [20-24]. In this study, we observed that *PRRT2* mutations are also common in Chinese families with BFIE and ICCA.

Including our mutations, 62 different *PRRT2* mutations have been described in literature in B(F)IE (Additional file 2: Table S2). To date, 170 BFIE families and 32 sporadic BIE cases have been screened for *PRRT2* mutations. *PRRT2* mutations were identified in 74.1% (126/170) of BFIE families and 34.4% (11/32) of sporadic BIE cases (Additional file 3: Table S3). Fifteen different *PRRT2* mutations were identified in BFIE or BIE (Additional file 2: Table S2), including two insertion mutations, five deletion mutations, four missense mutations, one nonsense mutation, and three splice site mutations. References cited in Additional file 2: Table S2 and Additional file 3: Table S3 were listed in the Additional file 4: Supplemental references. The mutation rates of *PRRT2* in BFIE ranged from 54.5% to 85.7% in previous studies [6,12,17].

Our study provides the first report of the deletion mutation c.649delC and the insertion mutation c.904_905insG being associated with a phenotype of BFIE only. The mutation c.649delC has been previously reported in PKD, ICCA, and HM [13,22,25-27], but not in families with BFIE. This mutation was observed in our three BFIE families (Families 10–12). The mutation c.904_905insG was previously described in one ICCA family [21]. We detected this mutation in one BFIE family (Family 13). The most described mutation c.649_650insC is a hotspot for *PRRT2* mutation. This was also present in our BFIE families, accounting for 69.2% (9/13) of *PRRT2* mutation-positive families. In previous studies, the percentage of c.649_650insC in BFIE families with a *PRRT2* mutation ranged from 85.7% to 92.9% [6,12,24]. This mutation was observed mainly with a BFIE, PKD and ICCA phenotype [6,8,12,13,28,29], and but also in a few familial or sporadic cases of PNKD, PED, HM and episodic ataxia [20-22]. This site appears to be particularly prone to mutation: it occurs in a tract of nine cytosines preceded by four guanines, facilitating the formation of a hairpin loop and slippage during DNA replication. Besides the deletion mutation c.649delC and the insertion mutation c.649_650insC, a nonsense mutation c.649C > T affecting the same nucleotide has been reported [13,15,25,30-33]. Incomplete penetrance was found in five of our BFIE families (Families 4, 5, 6, 8 and13). This phenomenon has also been described in other studies [6,12,28]. It was reported that a penetrance of *PRRT2* mutations in BFIE was 82% [12].

In our study, two probands of BFIE families experienced a single diarrhea-induced seizure. We cannot differentiate whether the late relapse of seizures in these two patients is a manifestation of convulsions with mild gastroenteritis (CwG), or the presentation of BFIE itself. CwG is a well-recognized infant seizure disorder associated with mild diarrhea. It is characterized by [34]: (1) previously healthy infants and young children aged 6 months to 3 years having afebrile generalized convulsions associated with symptoms of gastroenteritis; (2) seizures occuring sometimes in cluster; (3) normal laboratory examination results including electrolytes, blood glucose and cerebrospinal fluid; (4) normal interictal electroencephalography; and (5) excellent seizure and developmental outcomes. The incidence of CwG is thought to be higher in Asian populations than in people from Western countries [35-38]. Okumura reported that approximately 10% of children with BIE experienced CwG [39]. To explore whether *PRRT2* mutations are associated with CwG, several groups conducted *PRRT2* mutation analysis in patients with CwG, but no *PRRT2* mutation was found [18,24,40].

In our study, *PRRT2* mutations were present in 59.1% of Chinese BFIE families, presumably relating to the ethnic background. For the absence of a mutation in *PRRT2* in our remaining nine BFIE families, microdeletion of the *PRRT2* gene or other BFIE-related genes (*SCN2A*, *KCNQ2*, and *KCNQ3*) should be to be screened [29]. Microdeletions of *PRRT2* gene have been found in sporadic cases of PKD and ICCA [41], and also in one PKD family [33].

Including our ICCA families, 95 families with ICCA has been reported (Additional file 3: Table S3). 23 different *PRRT2* mutations have been reported in the ICCA phenotype (Additional file 2: Table S2): seven insertion mutations, four deletion mutations, four missense mutations, six nonsense mutations, one splice site mutation, and one microdeletion. Overall, *PRRT2* is reported to be mutated in 83.3%-100% of ICCA families [6,8,18,31], although one study reported that the much lower rate of 37.5% [17].We reviewed the *PRRT2* mutation rate in BFIE and ICCA families described in literature and our study (Additional file 3: Table S3), and found that it was higher (91.6%, 87/95) in ICCA families than in BFIE families (74.1%, 126/170).

The mutation c.514-517delTCTG has been previously observed in one family with PKD [5], and we found this mutation in an ICCA family. The mutation c.649delC has been reported in one sporadic ICCA case. In our study, we found three BFIE (Families 10–12) and three ICCA families (Families 26–28) with this mutation. This suggests that this mutation is the second most common mutation in Chinese families with BFIE and ICCA. We identified a novel stop codon mutation c.1023A > T (X341C) in one ICCA family. As far as we know, this mutation is the first reported mutation of *PRRT2* exon 4. It is predicted to change the stop codon into a cysteine, and would introduce a new stop codon after a 28-amino acids elongation of the C-terminal tail, which forms the extracellular domain of the PRRT2 protein. Functional studies of this mutation should be performed in the future.

We observed some uncommon phenotypes in our ICCA families. Family 23 is an ICCA family with c.649_650insC mutation. In this family, migraine co-existed with ICCA in one individual and with PKD in another. Both relatives had the 649_650inC mutation. The phenotype of migraine (with or without aura) has been reported in BFIE, PKD or ICCA families with a *PRRT2* mutation [17,19,20,22,33,42,43]. *PRRT2* mutation was identified in one large family with isolated HM [20]. The reported frequency of migraine among *PRRT2* mutation carriers is significantly higher (27.1%) than in the overall population with epilepsy (8%-15%) [19,44]. Therefore, the association of migraine with *PRRT2* mutation may not be coincidental. In Family 27, the proband (III-4) with ICCA also had an episode of febrile seizure (FS) at 7 years old, conforming to the diagnosis of febrile seizures plus (FS+). Patients with FS or FS + have been reported in families with BFIE [12,23,29,40,45], PKD [25,30,46] or ICCA [15,17,18]. Some carried *PRRT2* gene mutations. However, FS may not be associated with *PRRT2* mutations in ICCA families. For example, *PRRT2* mutation did not co-segregate with FS in a large ICCA family [15]. In a previously described ICCA family, only two of the four FS patients had a *PRRT2* mutation [18]. FS are more common in patients with epilepsy than in the general population [47]. It is therefore plausible that the occurrence of febrile seizures in patients with a *PRRT2* gene mutation is not solely caused by this mutation, but by the occurrence of epilepsy itself. It could also be caused by a mutation in another gene. The proband in Family 28 had PED. In ICCA families, paroxysmal dyskinesias are mostly of the kinesigenic type, but families with PED have also been reported [48]. A phenotype of PED with *PRRT2* mutation has been reported in two Chinese ICCA families [21].

## Conclusions

In summary, we confirm that *PRRT2* is the major causative gene for BFIE and ICCA in Chinese families. Site c.649 is a mutation hotspot: c.649_650insC is the most common mutation, and c.649delC is the second most common mutation in Chinese families with BFIE and ICCA. We also report a novel mutation, c.1023A > T (X341C), in one ICCA family. Migraine with aura and febrile seizures plus could co-exist in ICCA families.

## Abbreviations

BFIE: Benign familial infantile epilepsy; ICCA: Infantile convulsions with paroxysmal choreoathetosis; PKD: Paroxysmal kinesigenic dyskinesia; PKC: Paroxysmal kinesigenic choreoathetosis; EEG: Electroencephalogram; CwG: Benign convulsions with mild gastroenteritis; PNKD: Paroxysmal non-kinesigenic dyskinesia; PED: Paroxysmal exertion-induced dyskinesia; HM: Hemiplegic migraine; BIE: Benign infantile epilepsy; EA: Episodic ataxia; FS+: Febrile seizures plus; FS: Febrile seizures.

## Competing interests

The authors declare that they have no competing interests.

## Authors’ contributions

YZ designed the study and contributed to the initial draft of the manuscript. YZ, XX, SW, ZY, YW, XL and XW assessed the patients clinically, performed the phenotyping, and collected the DNA samples. XY extracted DNA from peripheral blood, analyzed the clinical and genetic data, and drafted the manuscript. All authors read and approved the final manuscript.

## Pre-publication history

The pre-publication history for this paper can be accessed here:

http://www.biomedcentral.com/1471-2377/13/209/prepub

## Supplementary Material

Additional file 1: Table S1 Clinical features and *PRRT2* mutations in the 73 affected relatives from 22 BFIE families. Click here for file

Additional file 2: Table S2 Reported *PRRT2* mutations allocated to the different phenotypes of BFIE, PKD, ICCA and others. Click here for file

Additional file 3: Table S3 Reported familial or sporadic cases screened *PRRT2* gene in different phenotypes. Click here for file

Additional file 4: Supplemental references Click here for file
