# Supplementary material for: Phenotypes and PRRT2 mutations in Chinese families with benign familial infantile epilepsy and infantile convulsions with paroxysmal choreoathetosis
Source: BMC Neurol. 2013 Dec 26;13:209. doi: 10.1186/1471-2377-13-209 (PMC3897939; doi:10.1186/1471-2377-13-209)
Supplement: Additional file 1: Table S1 — Clinical features and PRRT2 mutations in the 73 affected relatives from 22 BFIE families. [file 1471-2377-13-209-S1.doc]

Additional file1: Table S1

Table S1. Clinical features and *PRRT2* mutations in the 73 affected relatives from 22 BFIE families

| Family  No | Affected member | Age of seizure onset(m) | Age of  remission(m) | *PRRT2* mutation |
| --- | --- | --- | --- | --- |
| 1 | IV-15 | 6 | 13 | c.649_650insC |
|  | III-3 | <12 | <12 | c.649_650insC |
|  | I-1 | <12 | <24 | na |
|  | II-1 | <12 | <24 | na |
|  | III-2 | <12 | <24 | na |
|  | III-7 | <12 | <24 | na |
|  | IV-1 | 6 | <12 | na |
|  | IV-4 | <6 | <12 | na |
|  | IV-5 | 6 | <12 | na |
|  | IV-7 | <6 | <12 | na |
|  | IV-18 | 6 | <12 | na |
|  | V-1 | 6 | 7 | na |
|  | V-2 | 6 | 7 | na |
|  | V-4 | 6 | <12 | na |
|  | V-5 | 6 | 11 | na |
|  | V-6 | <12 | <12 | na |
|  | V-8 | 6 | <12 | na |
|  | V-9 | <6 | <12 | na |
| 2 | IV-6 | 5 | 6 | c.649_650insC |
|  | IV-9 | 2 | na | c.649_650insC |
|  | III-9 | <12 | <12 | c.649_650insC |
|  | II-3 | <12 | <12 | c.649_650insC |
|  | II-5 | <12 | <12 | na |
|  | IV-1 | <12 | <24 | na |
|  | IV-4 | <12 | <24 | na |
| 3 | IV-8 | 4 | 5 | c.649_650insC |
|  | III-12 | <12 | 12 | c.649_650insC |
|  | II-3 | <12 | <24 | na |
|  | III-7 | <12 | <12 | na |
|  | III-9 | <12 | <24 | na |
|  | III-10 | <12 | <12 | na |
|  | III-14 | <12 | <24 | na |
|  | IV-5 | <12 | <24 | na |
|  | IV-7 | <12 | <24 | na |
| 4 | III-6 | 5 | 6 | c.649_650insC |
| 5 | III-2 | 6 | 12 | c.649_650insC |
| 6 | III-6 | 6 | 12 | c.649_650insC |
|  | III-1 | <12 | 12 | na |
|  | III-4 | <12 | 18 | na |
|  | II-1 | <12 | <24 | na |
| 7 | III-3 | 2 | 3 | c.649_650insC |
|  | II-4 | 6 | 12 | c.649_650insC |
| 8 | II-1 | <12 | 12 | na |
| 9 | II-1 | 5 | <24 | c.649_650insC |
|  | II-3 | 5 | <24 | na |
| 10 | II-1 | 4.5 | 10 | c.649delC |
| 11 | II-1 | <12 | <24 | c.649delC |
|  | II-4 | <12 | <24 | na |
| 12 | II-1 | 1 | 5 | c.649delC |
| 13 | II-1 | 3 | 6 | na |
| 14 | II-2 | <12 | 12 |  |
|  | II-3 | <12 | 12 |  |
|  | II-5 | <12 | 12 |  |
|  | II-8 | <12 | 12 |  |
|  | II-10 | <12 | 12 |  |
|  | III-7 | <12 | 12 |  |
|  | III-9 | <12 | 12 |  |
|  | III-11 | <12 | 12 |  |
|  | III-15 | <12 | 12 |  |
|  | III-17 | <12 | 12 |  |
|  | IV-7 | <12 | 12 |  |
| 15 | II-1 | 3 | 4 |  |
| 16 | II-6 | 6 | 7 |  |
| 17 | II-2 | 3 | 24 |  |
|  | III-1 | 4 | 6 |  |
| 18 | II-1 | 2 | 6 |  |
| 19 | II-1 | 3 | 4 |  |
| 20 | II-5 | <12 | 12 |  |
| 21 | II-2 | <12 | 24 |  |
|  | III-2 | <12 | 24 |  |
|  | III-3 | <12 | 24 |  |
| 22 | III-1 | 8 | 18 |  |
|  | II-1 | <12 | <24 |  |

m: months, na: not available, Families 1–13 are *PRRT2* mutation-positive, and Families 14–22 are *PRRT2* mutation-negative.
