# Supplementary material for: Phenotypes and PRRT2 mutations in Chinese families with benign familial infantile epilepsy and infantile convulsions with paroxysmal choreoathetosis
Source: BMC Neurol. 2013 Dec 26;13:209. doi: 10.1186/1471-2377-13-209 (PMC3897939; doi:10.1186/1471-2377-13-209)
Supplement: Additional file 4: Supplemental references [file 1471-2377-13-209-S4.doc]

Additional file 4: Supplementary references

References cited for Additional file 2: Table S2 and Additional file 3: Table S3 were shown in below.

1. Chen WJ, Lin Y, Xiong ZQ, Wei W, Ni W, Tan GH, Guo SL, He J, Chen YF, Zhang QJ, Li HF, Lin Y, Murong SX, Xu J, Wang N, Wu ZY: **Exome sequencing identifies truncating mutations in PRRT2 that cause paroxysmal kinesigenic dyskinesia.** *Nat Genet* 2011, **43:**1252-1255.

2. Wang JL, Cao L, Li XH, Hu ZM, Li JD, Zhang JG, Liang Y, San A, Li N, Chen SQ, Guo JF, Jiang H, Shen L, Zheng L, Mao X, Yan WQ, Zhou Y, Shi YT, Ai SX, Dai MZ, Zhang P, Xia K, Chen SD, Tang BS: **Identification of PRRT2 as the causative gene of paroxysmal kinesigenic dyskinesias.** *Brain* 2011, **134:**3493-3501.

3. Li J, Zhu X, Wang X, Sun W, Feng B, Du T, Sun B, Niu F, Wei H, Wu X, Dong L, Li L, Cai X, Wang Y, Liu Y: **Targeted genomic sequencing identifies PRRT2 mutations as a cause of paroxysmal kinesigenic choreoathetosis.** *J Med Genet* 2012, **49:**76-78.

4. Liu Q, Qi Z, Wan XH, Li JY, Shi L, Lu Q, Zhou XQ, Qiao L, Wu LW, Liu XQ, Yang W, Liu Y, Cui LY, Zhang X: **Mutations in PRRT2 result in paroxysmal dyskinesias with marked variability in clinical expression.** *J Med Genet* 2012, **49:**79-82.

5. Heron SE, Grinton BE, Kivity S, Afawi Z, Zuberi SM, Hughes JN, Pridmore C, Hodgson BL, Iona X, Sadleir LG, Pelekanos J, Herlenius E, Goldberg-Stern H, Bassan H, Haan E, Korczyn AD, Gardner AE, Corbett MA, Gecz J, Thomas PQ, Mulley JC, Berkovic SF, Scheffer IE, Dibbens LM: **PRRT2 mutations cause benign familial infantile epilepsy and infantile convulsions with choreoathetosis syndrome.** *Am J Hum Genet* 2012, **90:**152-160.

6. Lee HY, Huang Y, Bruneau N, Roll P, Roberson ED, Hermann M, Quinn E, Maas J, Edwards R, Ashizawa T, Baykan B, Bhatia K, Bressman S, Bruno MK, Brunt ER, Caraballo R, Echenne B, Fejerman N, Frucht S, Gurnett CA, Hirsch E, Houlden H, Jankovic J, Lee WL, Lynch DR, Mohammed S, Muller U, Nespeca MP, Renner D, Rochette J, et al: **Mutations in the gene PRRT2 cause paroxysmal kinesigenic dyskinesia with infantile convulsions.** *Cell Rep* 2012, **1:**2-12.

7. Cao L, Huang XJ, Zheng L, Xiao Q, Wang XJ, Chen SD: **Identification of a novel PRRT2 mutation in patients with paroxysmal kinesigenic dyskinesias and c.649dupC as a mutation hot-spot.** *Parkinsonism Relat Disord* 2012, **18:**704-706.

8. Dale RC, Grattan-Smith P, Nicholson M, Peters GB: **Microdeletions detected using chromosome microarray in children with suspected genetic movement disorders: a single-centre study.** *Dev Med Child Neurol* 2012, **54:**618-623.

9. Ono S, Yoshiura K, Kinoshita A, Kikuchi T, Nakane Y, Kato N, Sadamatsu M, Konishi T, Nagamitsu S, Matsuura M, Yasuda A, Komine M, Kanai K, Inoue T, Osamura T, Saito K, Hirose S, Koide H, Tomita H, Ozawa H, Niikawa N, Kurotaki N: **Mutations in PRRT2 responsible for paroxysmal kinesigenic dyskinesias also cause benign familial infantile convulsions.** *J Hum Genet* 2012, **57:**338-341.

10. Schubert J, Paravidino R, Becker F, Berger A, Bebek N, Bianchi A, Brockmann K, Capovilla G, Dalla Bernardina B, Fukuyama Y, Hoffmann GF, Jurkat-Rott K, Anttonen AK, Kurlemann G, Lehesjoki AE, Lehmann-Horn F, Mastrangelo M, Mause U, Muller S, Neubauer B, Pust B, Rating D, Robbiano A, Ruf S, Schroeder C, Seidel A, Specchio N, Stephani U, Striano P, Teichler J, et al: **PRRT2 mutations are the major cause of benign familial infantile seizures.** *Hum Mutat* 2012, **33:**1439-1443.

11. Groffen AJ, Klapwijk T, van Rootselaar AF, Groen JL, Tijssen MA: **Genetic and phenotypic heterogeneity in sporadic and familial forms of paroxysmal dyskinesia.** *J Neurol* 2013, **260:**93-99.

12. Steinlein OK, Villain M, Korenke C: **The PRRT2 mutation c.649dupC is the so far most frequent cause of benign familial infantile convulsions.** *Seizure* 2012, **21:**740-742.

13. Meneret A, Grabli D, Depienne C, Gaudebout C, Picard F, Durr A, Lagroua I, Bouteiller D, Mignot C, Doummar D, Anheim M, Tranchant C, Burbaud P, Jedynak CP, Gras D, Steschenko D, Devos D, Billette de Villemeur T, Vidailhet M, Brice A, Roze E: **PRRT2 mutations: a major cause of paroxysmal kinesigenic dyskinesia in the European population.** *Neurology* 2012, **79:**170-174.

14. Specchio N, Terracciano A, Trivisano M, Cappelletti S, Claps D, Travaglini L, Cusmai R, Marras CE, Zara F, Fusco L, Bertini E, Vigevano F: **PRRT2 is mutated in familial and non-familial benign infantile seizures.** *Eur J Paediatr Neurol* 2013, **17:**77-81.

15. Wang K, Zhao X, Du Y, He F, Peng G, Luo B: **Phenotypic overlap among paroxysmal dyskinesia subtypes: Lesson from a family with PRRT2 gene mutation.** *Brain Dev* 2012.

16. Dale RC, Gardiner A, Antony J, Houlden H: **Familial PRRT2 mutation with heterogeneous paroxysmal disorders including paroxysmal torticollis and hemiplegic migraine.** *Dev Med Child Neurol* 2012, **54:**958-960.

17. Lee YC, Lee MJ, Yu HY, Chen C, Hsu CH, Lin KP, Liao KK, Chang MH, Liao YC, Soong BW: **PRRT2 mutations in paroxysmal kinesigenic dyskinesia with infantile convulsions in a Taiwanese cohort.** *PLoS One* 2012, **7:**e38543.

18. van Vliet R, Breedveld G, de Rijk-van Andel J, Brilstra E, Verbeek N, Verschuuren-Bemelmans C, Boon M, Samijn J, Diderich K, van de Laar I, Oostra B, Bonifati V, Maat-Kievit A: **PRRT2 phenotypes and penetrance of paroxysmal kinesigenic dyskinesia and infantile convulsions.** *Neurology* 2012, **79:**777-784.

19. Friedman J, Olvera J, Silhavy JL, Gabriel SB, Gleeson JG: **Mild paroxysmal kinesigenic dyskinesia caused by PRRT2 missense mutation with reduced penetrance.** *Neurology* 2012, **79:**946-948.

20. Cai C, Shi O, Li WD: **Missense mutations of the proline-rich transmembrane protein 2 gene cosegregate with mild paroxysmal kinesigenic dyskinesia and infantile convulsions in a Chinese pedigree.** *Parkinsonism Relat Disord* 2013, **19:**402-403.

21. Labate A, Tarantino P, Viri M, Mumoli L, Gagliardi M, Romeo A, Zara F, Annesi G, Gambardella A: **Homozygous c.649dupC mutation in PRRT2 worsens the BFIS/PKD phenotype with mental retardation, episodic ataxia, and absences.** *Epilepsia* 2012, **53:**e196-199.

22. Ishii A, Yasumoto S, Ihara Y, Inoue T, Fujita T, Nakamura N, Ohfu M, Yamashita Y, Takatsuka H, Taga T, Miyata R, Ito M, Tsuchiya H, Matsuoka T, Kitao T, Murakami K, Lee WT, Kaneko S, Hirose S: **Genetic analysis of PRRT2 for benign infantile epilepsy, infantile convulsions with choreoathetosis syndrome, and benign convulsions with mild gastroenteritis.** *Brain Dev* 2013, **35:**524-530.

23. Okumura A, Shimojima K, Kubota T, Abe S, Yamashita S, Imai K, Okanishi T, Enoki H, Fukasawa T, Tanabe T, Dibbens LM, Shimizu T, Yamamoto T: **PRRT2 mutation in Japanese children with benign infantile epilepsy.** *Brain Dev* 2012.

24. Li HF, Ni W, Xiong ZQ, Xu J, Wu ZY: **PRRT2 c.649dupC mutation derived from de novo in paroxysmal kinesigenic dyskinesia.** *CNS Neurosci Ther* 2013, **19:**61-65.

25. Sheerin UM, Stamelou M, Charlesworth G, Shiner T, Spacey S, Valente EM, Wood NW, Bhatia KP: **Migraine with aura as the predominant phenotype in a family with a PRRT2 mutation.** *J Neurol* 2013, **260:**656-660.

26. Scheffer IE, Grinton BE, Heron SE, Kivity S, Afawi Z, Iona X, Goldberg-Stern H, Kinali M, Andrews I, Guerrini R, Marini C, Sadleir LG, Berkovic SF, Dibbens LM: **PRRT2 phenotypic spectrum includes sporadic and fever-related infantile seizures.** *Neurology* 2012, **79:**2104-2108.

27. Gardiner AR, Bhatia KP, Stamelou M, Dale RC, Kurian MA, Schneider SA, Wali GM, Counihan T, Schapira AH, Spacey SD, Valente EM, Silveira-Moriyama L, Teive HA, Raskin S, Sander JW, Lees A, Warner T, Kullmann DM, Wood NW, Hanna M, Houlden H: **PRRT2 gene mutations: from paroxysmal dyskinesia to episodic ataxia and hemiplegic migraine.** *Neurology* 2012, **79:**2115-2121.

28. de Vries B, Callenbach PM, Kamphorst JT, Weller CM, Koelewijn SC, ten Houten R, de Coo IF, Brouwer OF, van den Maagdenberg AM: **PRRT2 mutation causes benign familial infantile convulsions.** *Neurology* 2012, **79:**2154-2155.

29. Castiglioni C, Lopez I, Riant F, Bertini E, Terracciano A: **PRRT2 mutation causes paroxysmal kinesigenic dyskinesia and hemiplegic migraine in monozygotic twins.** *Eur J Paediatr Neurol* 2013, **17:**254-258.

30. Cloarec R, Bruneau N, Rudolf G, Massacrier A, Salmi M, Bataillard M, Boulay C, Caraballo R, Fejerman N, Genton P, Hirsch E, Hunter A, Lesca G, Motte J, Roubertie A, Sanlaville D, Wong SW, Fu YH, Rochette J, Ptacek LJ, Szepetowski P: **PRRT2 links infantile convulsions and paroxysmal dyskinesia with migraine.** *Neurology* 2012, **79:**2097-2103.

31. Marini C, Conti V, Mei D, Battaglia D, Lettori D, Losito E, Bruccini G, Tortorella G, Guerrini R: **PRRT2 mutations in familial infantile seizures, paroxysmal dyskinesia, and hemiplegic migraine.** *Neurology* 2012, **79:**2109-2114.

32. Riant F, Roze E, Barbance C, Meneret A, Guyant-Marechal L, Lucas C, Sabouraud P, Trebuchon A, Depienne C, Tournier-Lasserve E: **PRRT2 mutations cause hemiplegic migraine.** *Neurology* 2012, **79:**2122-2124.

33. Liu XR, Wu M, He N, Meng H, Wen L, Wang JL, Zhang MP, Li WB, Mao X, Qin JM, Li BM, Tang B, Deng YH, Shi YW, Su T, Yi YH, Tang BS, Liao WP: **Novel PRRT2 mutations in paroxysmal dyskinesia patients with variant inheritance and phenotypes.** *Genes Brain Behav* 2013, **12:**234-240.

34. Hedera P, Xiao J, Puschmann A, Momcilovic D, Wu SW, LeDoux MS: **Novel PRRT2 mutation in an African-American family with paroxysmal kinesigenic dyskinesia.** *BMC Neurol* 2012, **12:**93.

35. Becker F, Schubert J, Striano P, Anttonen AK, Liukkonen E, Gaily E, Gerloff C, Muller S, Heussinger N, Kellinghaus C, Robbiano A, Polvi A, Zittel S, von Oertzen TJ, Rostasy K, Schols L, Warner T, Munchau A, Lehesjoki AE, Zara F, Lerche H, Weber YG: **PRRT2-related disorders: further PKD and ICCA cases and review of the literature.** *J Neurol* 2013, **260:**1234-1244.

36. Labate A, Tarantino P, Palamara G, Gagliardi M, Cavalcanti F, Ferlazzo E, Sturniolo M, Incorpora G, Annesi G, Aguglia U, Gambardella A: **Mutations in PRRT2 result in familial infantile seizures with heterogeneous phenotypes including febrile convulsions and probable SUDEP.** *Epilepsy Res* 2013, **104:**280-284.

37. Zara F, Specchio N, Striano P, Robbiano A, Gennaro E, Paravidino R, Vanni N, Beccaria F, Capovilla G, Bianchi A, Caffi L, Cardilli V, Darra F, Bernardina BD, Fusco L, Gaggero R, Giordano L, Guerrini R, Incorpora G, Mastrangelo M, Spaccini L, Laverda AM, Vecchi M, Vanadia F, Veggiotti P, Viri M, Occhi G, Budetta M, Taglialatela M, Coviello DA, et al: **Genetic testing in benign familial epilepsies of the first year of life: clinical and diagnostic significance.** *Epilepsia* 2013, **54:**425-436.

38. van Strien TW, van Rootselaar AF, Hilgevoord AA, Linssen WH, Groffen AJ, Tijssen MA: **Paroxysmal kinesigenic dyskinesia: cortical or non-cortical origin.** *Parkinsonism Relat Disord* 2012, **18:**645-648.

39. Schmidt A, Kumar KR, Redyk K, Grunewald A, Leben M, Munchau A, Sue CM, Hagenah J, Hartmann H, Lohmann K, Christen HJ, Klein C: **Two faces of the same coin: benign familial infantile seizures and paroxysmal kinesigenic dyskinesia caused by PRRT2 mutations.** *Arch Neurol* 2012, **69:**668-670.

40. Silveira-Moriyama L, Gardiner AR, Meyer E, King MD, Smith M, Rakshi K, Parker A, Mallick AA, Brown R, Vassallo G, Jardine PE, Guerreiro MM, Lees AJ, Houlden H, Kurian MA: **Clinical features of childhood-onset paroxysmal kinesigenic dyskinesia with PRRT2 gene mutations.** *Dev Med Child Neurol* 2013, **55:**327-334.

41. Shi CH, Sun SL, Wang JL, Liu AQ, Miao W, Avinash C, Mao X, Tang BS, Xu YM: **PRRT2 gene mutations in familial and sporadic paroxysmal kinesigenic dyskinesia cases.** *Mov Disord* 2013.

42. Chen YP, Song W, Yang J, Zheng ZZ, Huang R, Chen K, Zhao B, Chen XP, Burgunder JM, Shang HF: **PRRT2 mutation screening in patients with paroxysmal kinesigenic dyskinesia from Southwest China.** *Eur J Neurol* 2013.

43. Jing XY, Li XH, Yuan P, Deng J, Hu B, Wang Y: **A novel mutation and functional implications of 5 variants in the PRRT2 gene in 20 paroxysmal kinesigenic dyskinesia pedigrees.** *Parkinsonism Relat Disord* 2013.

44. Lambrecq V, Riant F, Tournier-Lasserve E, Michel V, Burbaud P: **Caffeine improved paroxysmal dyskinesia caused by the PRRT2 mutation.** *Mov Disord* 2013.

45. Tan LC, Methawasin K, Teng EW, Ng AR, Seah SH, Au WL, Liu JJ, Foo JN, Zhao Y, Tan EK: **Clinico-genetic comparisons of paroxysmal kinesigenic dyskinesia patients with and without PRRT2 mutations.** *Eur J Neurol* 2013.
